# An evaluation of junior doctors’ experience in smoking cessation training in a rural mental health setting

**DOI:** 10.3389/fpsyt.2022.868212

**Published:** 2022-08-25

**Authors:** Nicholas Faint, Beatriz Cuesta-Briand, Mathew Coleman

**Affiliations:** ^1^Great Southern Mental Health Service, Albany, WA, Australia; ^2^The Rural Clinical School of Western Australia, Albany, WA, Australia; ^3^Telethon Kids Institute, Perth Children’s Hospital, Nedlands, WA, Australia

**Keywords:** mental illness, tobacco cessation, rural, rural mental health, junior doctor education, smoking

## Abstract

**Introduction:**

Smoking prevalence remains high amongst people with mental illness, however, they are less likely to be screened for tobacco dependence and offered treatment to quit. Smoking cessation and education training are insufficient in medical schools, despite a positive relationship between training and practice once qualified. However, the question as to whether there is adequate skill and expertise to address smoking in people with mental illness within Australian mental health settings is unclear. Furthermore, people living in rural and remote areas smoke at higher rates, quit at lower rates than those in urban areas, and experience limitations in their ability to access smoking cessation supports. The Smokers’ Clinic is an initiative established in a rural Australian mental health service offering a smoking cessation service to patients and staff employed by the service.

**Aim:**

This study aims to assess the change in the knowledge and confidence of resident medical officers in their understanding of nicotine dependence, smoking cessation strategies and prescribing nicotine replacement therapy in a community mental health setting. It was hypothesized that providing education and supervised clinical experience would improve knowledge, increasing confidence and motivation in managing smoking cessation in mental health patients. The research was undertaken using data collected through a questionnaire obtained from surveying resident medical officers administering the Smokers’ Clinic following a 10-week rural community mental health rotation.

**Materials and methods:**

Twenty resident medical officers completed the 10-week rotation, with 14 completing the questionnaire. Knowledge of tobacco smoking, nicotine dependence and smoking cessation interventions improved with the experience of the Smokers’ Clinic during the clinical rotation. Resident medical officers were motivated to spend additional time engaged in self-directed learning and all reported continued use of acquired experience and information in their clinical work after the rotation.

**Conclusion:**

This study indicates the utility of a novel approach in delivering education, training, building clinical expertise, and facilitating sustained clinical capacity amongst junior medical staff for smoking cessation in a rural community mental health setting. It offers an efficient approach for mental health services to deliver smoking cessation services to reduce the morbidity and mortality burden associated with tobacco smoking.

## Introduction

Smoking is the leading cause of preventable disease burden in Australia, and worldwide (1, 2). Economically, the net cost for smoking to Australian society was $136.9 billion in 2015–2016 (1). Although tobacco can be snorted and chewed, smoking is the predominant form of consumption in Australia (1). Across the greater Australian community, the prevalence of smoking continues to decline, however, it remains high among those with mental illness (3). The prevalence of adults smoking daily has declined from 12.8% in 2016 to 11.6% in 2019, however, it remains high at 20% in those with mental health conditions (1). People with mental illness have higher levels of nicotine dependence, lower rates of smoking cessation and consequently suffer from higher rates of morbidity associated with smoking compared to the general population (4). Prevalence rates are highest in those diagnosed with bipolar affective disorder who are three and a half times more likely to smoke than the general population, and schizophrenia who are more than five times as likely to smoke than the general population (3). They are also more likely to die from smoking-related illnesses, such as cardiovascular disease, respiratory disease and cancer, as opposed to their mental illness (5–11).

Public health interventions to reduce demand (advertising restrictions, plain packaging, mass educational campaigns, legislation restricting advertisement) (12), reduce harm (subsidization of pharmaceuticals to assist with cessation, legislation mandating smoke-free workplaces) (13), and reduce supply (tobacco taxation, restriction of sales to minors below 18 years of age) (12), have been effective in decreasing the prevalence of smoking in the Australian population. In recent years, many countries have prohibited patients from smoking in mental health facilities, or on hospital grounds. However, many of these interventions have had minimal effect on smoking rates in people with mental illnesses, likely due to the lack of strategic targeting to address distinct barriers to cessation in this population (14).

Tobacco smoking has long been embedded within mental health service culture: “smoking rooms” have acted as social hubs for patients; smoking itself has provided structure and social activity for patients and staff alike; and cigarettes have been used to both placate or engage with patients (15, 16). Historically, tobacco companies have supplied either free or low cost cigarettes to institutions and have actively blocked efforts to institute smoking bans (17). The most popular explanation for high smoking rates among people with mental illness is the self-medication hypothesis, which posits this as an attempt to manage negative symptoms due to underlying neurobiological deficits associated with mental illness, leading to cognitive impairment (18). Rates of smoking amongst mental health professionals remains high compared with other professions, perpetuating the embedding of tobacco smoking within mental health culture (19).

With respect to accessing smoking cessation interventions, people with mental illness are less likely to be screened for tobacco dependence and offered treatment to assist in quitting (20). Mental health professionals hold attitudes and misconceptions that may undermine the delivery of effective smoking cessation interventions (21, 22). Myths surrounding smoking cessation for people with mental illness continue to persist. These include: people with mental illness are not motivated to quit smoking; smoking cessation is not possible for people with mental illness; smoking cessation is a lower priority for people with mental illness; smoking assists with stress; and smoking cessation is harmful to people with mental illness (10, 18, 23).

People with mental illness respond to interventions, and tolerate pharmaceutical interventions used to assist in cessation and abstinence from tobacco in the same way as those without mental illness (24, 25). The EAGLES trial, a study designed to evaluate the neuropsychiatric safety of Varenicline, Bupropion, nicotine patch, and placebo when used in smokers with and without mental illness, did not show a significant increase in neuropsychiatric adverse events and demonstrated efficacy of these pharmaceutical interventions in achieving smoking cessation (26). Moreover, people with mental illness prefer support and encouragement from mental health clinicians in their efforts to achieve abstinence from cigarettes, rather than accessing mainstream quit services (27). Smoking cessation in people with mental illness is associated with improvements in mental health, quality of life and reduction in other substance misuse (28). Mental health professionals are also perfectly placed to address the impact smoking cessation (and tobacco use) has on the metabolism of psychotropic medications (29).

Whilst overall, smoking rates in Australia have declined, these findings are not proportionate across geographical locations (30). People living in rural and remote areas smoke at higher rates and quit at lower rates compared to those in urban areas (30). Accessing community services is more difficult for rural and remote residents compared with urban residents, due to physical distance to services and social isolation. Rural and remote residents need to travel on average 90 min or 102.7 km in order to access healthcare supports (31). Travel times are often increased for Australian Aboriginal residents who are more likely to reside in very remote settings (31). Despite Australian government initiatives implemented to increase funding to rural and remote medical training, worryingly, medical workforce shortages and maldistributions between urban and rural and remote settings persist (32). Decreased availability of health professionals and decreased health expenditure both appear to correlate with increasing remoteness (33). Pro-tobacco social norms, lower socioeconomic and educational attainment, and different cultural attitudes are seen as significant contributing factors (30). Cumulatively, these factors may result in rural and remote residents experiencing limitations in their ability to access healthcare, maintain health beliefs that support cigarette smoking, and prevent access to smoking cessation medications and supports (30).

Countless opportunities are missed in addressing the disproportionately high prevalence rates of tobacco smoking in people with mental illness. A national survey of United Kingdom medical schools concluded that smoking cessation and education training was insufficient, and may have worsened over the preceding decade (34). This is despite research demonstrating retention of knowledge and skills among medical students who receive education on smoking assessment and interventions during medical school (35–37). A positive relationship exists between education received in medical school and consequential increases in knowledge, and the development of positive perceptions regarding role in initiating smoking cessation interventions for patients once qualified (38). However, the question as to whether there are adequate levels of skill and expertise to address smoking in people with mental illness within Australian mental health settings is unclear.

Previous studies have demonstrated that psychiatrists are less likely than general practitioners to advise people to quit smoking (39, 40). This may be due to reluctance in managing smoking cessation given a lack of evidence-based advice offering guidance for prescribing pharmacotherapies in people with mental illness, with low prescribing rates and utilization of behavioral interventions (18, 41, 42). In studies that led to United States Food and Drug Administration (FDA) approval for smoking cessation medications, people with mental illness were excluded. This lack of information has made it difficult for clinicians to manage smoking cessation in people with severe mental illness. It has also led to the non-use of these products, as clinicians fear they may not be safe (18, 43).

Nicotine replacement therapy (NRT) remains the mainstay of interventions offered to people with mental illnesses. This intervention is usually offered in the context of inpatient treatment and, invariably, with little attention to smoking cessation, but rather nicotine withdrawal management within non-smoking facilities. Training and education of mental health practitioners must be a priority in order to address the sustained high rates of tobacco smoking, morbidity and mortality in this vulnerable at-risk group.

## The Smokers’ Clinic

The Smokers’ Clinic is an initiative established in a rural Australian mental health service offering a smoking cessation service to patients (inpatient and outpatient) and staff employed by the service, based on the assessment protocol from the Brain Mind Research Institute (BMRI) at The University of Sydney (44). It offers clients an initial 1-h face-to-face assessment followed by weekly 30-min follow-up assessments (face-to-face, telephone, or video conference) for 6–8 weeks. The initial assessment consists of a comprehensive biopsychosocial history focused on the patient’s smoking history, allowing the implementation of a customized treatment plan. The clinic is accessible to patients and staff who utilize tobacco in all forms, along with e-cigarettes. It was established to meet an unmet need amongst this group of patients.

The Smokers’ Clinic is administered and conducted by a resident medical officer (RMO) who is undertaking a 10-week community mental health rotation within the service. As a junior medical practitioner, their experience of mental health settings, presentations and interventions is limited. The RMO receives an initial 1-h education session provided by their supervisor, an addiction consultant psychiatrist, in addition to ongoing weekly supervision to discuss issues related to tobacco smoking, nicotine dependence and treatment which includes pharmacotherapy and non-pharmacotherapy options. The Smokers’ Clinic is provided in parallel to the patient accessing mental health treatment as usual from the service. This initiative is advantageous as it offers mental health patients concurrent management of both mental health and substance use disorders. RMOs work in close collaboration with the patient’s treating team, assisting in the assessment of other substance use disorders. The Smokers’ Clinic also allows medication reviews to occur, as psychotropic medication dosages may need to be altered, due to drug interactions and metabolic changes that occur in the context of smoking cessation (25).

All clients undergo a comprehensive initial assessment including: standardized history and examination consistent with the BMRI protocol (44); the Fagerstrom Test for Nicotine Dependence (FTND) – an instrument that provides universally accepted detailed measure of nicotine dependence (low, low-moderate, moderate, high) in people with and without mental illness, to guide interventions (45, 46); and a Carboxymeter reading measuring expired Carbon Monoxide (eCO) levels. eCO levels can be used to confirm smoking status, make comparisons throughout follow up, and confirm abstinence. Correspondence pertaining to the patient’s progress is provided to their general practitioner and treating psychiatrist. Pharmacotherapies including combination NRT, varenicline, bupropion and nortriptyline are offered and were provided via prescription or available to purchase at a discounted rate from local pharmacies. Behavioral interventions such as individual counseling, motivational interviewing, and mindfulness-based strategies are utilized and incorporated into patients’ treatment plans. These are derived from and consistent with the Royal Australian College of General Practitioners (RACGP) Clinical Guidelines for Smoking Cessation (9). Quitline referral was offered to all clients.

## Aim

The aim of this study was to assess the change in the level of knowledge and confidence of RMOs in their understanding of nicotine dependence, smoking cessation strategies and prescribing NRT in a community mental health setting. This includes assessing their experience of their initial training and supervision from an addiction consultant psychiatrist during their 10-week rotation. It is hypothesized that providing *in situ* education and supervised clinical experience would result in an improvement in knowledge, increasing confidence and motivation in managing smoking cessation in mental health patients.

## Materials and methods

The research was undertaken using largely quantitative measures with two open-ended questions, obtained from a brief survey of RMOs administering the Smokers’ Clinic whilst undertaking a community mental health rotation. RMOs who undertook the rotation between 2016 and 2021 were sent an email at the conclusion of their rotation, containing information on the project and a hyperlink to complete an anonymous 19-question online questionnaire. Consent was assumed if the RMO completed the questionnaire. RMOs who completed more than one rotation were invited to complete the questionnaire only once. The questionnaire, developed by the investigators, recorded knowledge and confidence in the assessment and management of smoking cessation, knowledge, and confidence in relation to specific treatments and the applicability of this knowledge beyond the Smokers’ Clinic. The knowledge domains assessed were smoking, smoking cessation, and NRT. Results were recorded on five-point Likert-scales. For example, knowledge was assessed with “1” correlating with “none,” and “5” correlating with “excellent,” and for confidence “1” correlated with “not confident at all,” and “5” correlating with “very confident.” RMO knowledge was assessed before and after completion of the rotation, whilst confidence was assessed after completion of the rotation. There were two open questions where RMOs were invited to input a free text response. The questions asked RMOs to elaborate further on additional training they may have undergone, and to provide any additional comments at the conclusion of the questionnaire (the questionnaire administered can be provided upon request).

Descriptive statistics were used to summarize findings. A paired-samples *t*-test was used to compare RMO knowledge before and after completing their rotation operating the Smokers’ Clinic, with statistical significance set at *p* < 0.05. A basic content analysis was conducted on the responses to the two open-ended questions. Ethics approval was obtained through the Western Australia Country Health Service (WACHS) Health Research Ethics Committee (approval number RGS230).

## Results

A total of 20 RMOs completed a 10-week rotation in the community mental health setting over a 5-year period and were responsible for operating the Smokers’ Clinic. Of these 14 completed the questionnaire (70% response rate). At the time of completing the survey, three (21%) RMOs were undertaking their second postgraduate year (PGY), two (14%) were undertaking their third PGY, and the remaining nine (64%) had greater than 3 years experience.

Resident medical officers did not appear to encounter difficulty learning about *nicotine dependence* and *smoking cessation*, rating it as “very easy” (*n* = 5, 36% and *n* = 4, 29%, respectively), “easy” (*n* = 7, 50% and *n* = 7, 50%, respectively), or “moderate” (*n* = 2, 14% and *n* = 3, 21%, respectively). All RMOs spent additional time acquiring knowledge outside of the education provided by the addiction consultant psychiatrist with most reporting an additional 1–2 h (*n* = 7, 50%) of self-directed learning.

A variety of case complexity was experienced during clinic encounters with the majority of RMOs (*n* = 8, 57%) reporting moderate patient complexity. The majority of RMOs rated the amount of supervision from the addiction consultant psychiatrist as appropriate (*n* = 13, 93%).

**FIGURE 1:**
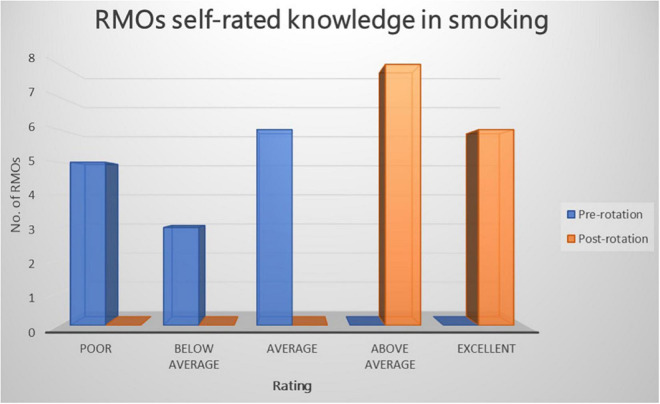
Graph illustrating the RMOs rated knowledge of smoking pre-rotation and post rotation in a community mental health setting using a five-point Likert-scale.

**FIGURE 2:**
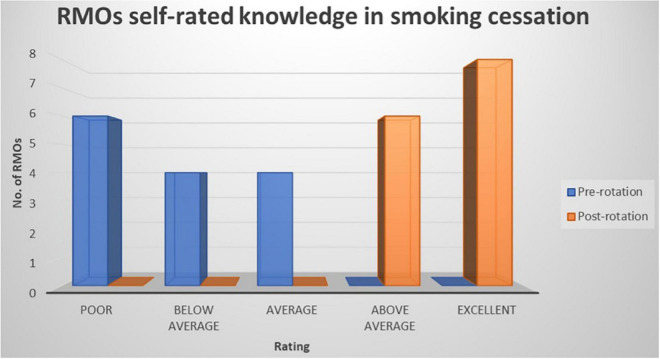
Graph illustrating the RMOs rated knowledge of smoking cessation pre-rotation and post rotation in a community mental health setting using a five-point Likert-scale.

**FIGURE 3:**
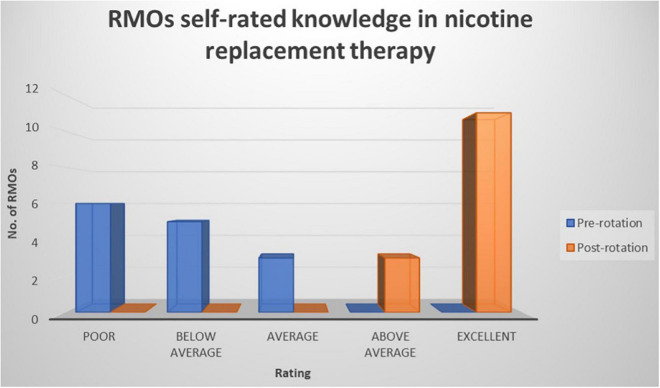
Graph illustrating the RMOs rated knowledge of nicotine replacement pre-rotation and post rotation in a community mental health setting using a five-point Likert-scale.

Prior to the commencement of the rotation, all RMOs rated their knowledge of smoking, smoking cessation, and NRT as “poor,” “below average,” or “average,” whereas after the rotation RMOs rated their knowledge as “above average” or “excellent” (refer to Figures 1–3). The improvement in knowledge for RMOs operating the Smokers’ Clinic was statistically significant for smoking (*t* = −17.73, *p* < 0.001), smoking cessation (*t* = −21.66, *p* < 0.001), and NRT (*t* = −16.52, *p* < 0.001) (refer to Table 1).

**TABLE 1:** Paired *t*-test comparing RMO rated knowledge of smoking, smoking cessation, and NRT before and after completing a community mental health rotation.

Knowledge domain	Pre-rotation M (SD) *N* = 14	Post-rotation M (SD) *N* = 14	*t*	*P*-value
Smoking	2.07 (0.92)	4.42 (0.86)	**−17.73**	**<0.001**
Smoking cessation	1.86 (0.86)	4.57 (0.51)	**−21.66**	**<0.001**
NRT	1.79 (0.80)	4.79 (0.43)	**−16.523**	**<0.001**

Values in bold indicate statistically significant results.

All RMOs have continued to use the acquired information for clinical work beyond the “Smokers’ Clinic” with the majority reporting use on a weekly basis (*n* = 8, 57%). All RMOs agreed they had a duty of care to advise and aid patients in their efforts to cut back and/or quit smoking. The majority of RMOs (*n* = 11, 79%) had not received any further training in smoking cessation or nicotine dependence beyond that experienced from the Smokers’ Clinic. Those RMOs who had further training were asked to elaborate – further training experiences were General Practitioner (GP) fellowship training, non-specific fellowship training, self-directed study, or conference presentations.

**FIGURE 4:**
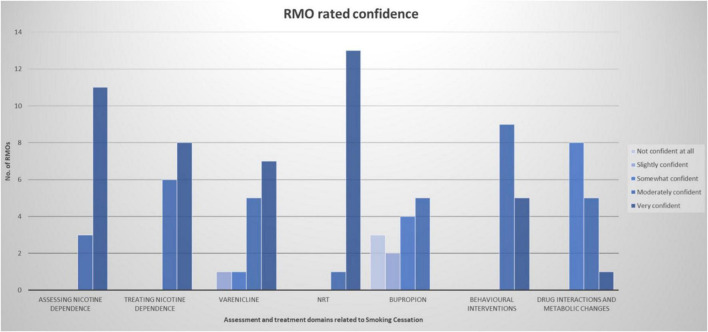
Graph illustrating the self-rated confidence of RMOs in various assessment and treatment domains related to smoking cessation after completing their rotation operating the Smokers’ Clinic using a five-point Likert-scale.

At the completion of their rotation, the majority of RMOs (*n* = 11, 79%) rated themselves as “very confident” in assessing for nicotine dependence, with all rating their confidence in administering treatment of nicotine dependence and smoking cessation as “moderately confident” (*n* = 6, 43%) or “very confident” (*n* = 8, 57%). When looking at “very confident” ratings for treatment interventions, NRT was the highest (*n* = 13, 93%), followed by Varenicline (*n* = 7, 50%), and behavioral interventions (*n* = 5, 36%). With respect to Bupropion, no RMO’s rated themselves as “very confident” with the majority (*n* = 5, 36%) rating themselves as moderately confident in prescribing and managing this agent. Regarding the recognition of drug interactions and changes in metabolism of psychotropic medication in the setting of smoking cessation in mental health patients, the majority of RMO’s (*n* = 8, 57%) rated themselves as “somewhat confident” with (*n* = 5, 36%) rating themselves as “moderately confident” and only one (*n* = 1, 7%) rating themselves as “very confident” (refer to Figure 4).

Resident medical officers were invited to provide additional feedback regarding their experience of the Smokers’ Clinic. Three comments were provided – two of which identified the educational/training benefits of the Smokers’ Clinic and the utility within their clinical practice, whilst one noted their lack of experience in using bupropion and indicated a necessity to further review drug interactions.

## Discussion

Junior doctors’ knowledge of tobacco smoking, nicotine dependence and smoking cessation interventions increased with the experience of the Smokers’ Clinic during their 10-week rotation. Furthermore, the relative ease at which learning occurred, the knowledge and clinical practice reported by RMOs are of significant educational and clinical importance. This is particularly the case in view of the relatively short experience and training resources required to enable this experience, and has significant implications in the context of a low resource rural setting. RMOs were exposed to a range of patient complexity which only serves to further contribute to building capacity within their training and confidence when assessing and managing mental health patients prescribed psychotropic medications. The Smokers’ Clinic structure and governance appeared to motivate RMOs to engage in their own self-directed learning outside of the provided education sessions.

Confidence in the assessment and management of smoking cessation by using NRT was high in this study. This is unsurprising given NRT is available for purchase without prescription (47), has proven safety over 30 years of use (48), and is recommended as first line pharmacological agents (9). Interestingly, study participants rated themselves as confident in prescribing Varenicline to patients with mental illnesses – contrasting with reports within the literature about psychiatrists’ attitudes toward this pharmacological agent for smoking cessation (26), and trends showing decreasing rates of Varenicline prescribing over the preceding 10 years (48). However, Bupropion was not used by most participants, and therefore, confidence ratings reflected the lack of exposure to this pharmacotherapy. This may also suggest that success, and dropout, meant that opportunities to progress to Bupropion did not occur. There may also be apprehension in prescribing due to reports within the literature of adverse effects (44). A variety of reasons may account for this finding, including the relatively short intervention period, a lack of continuity of patients over periods greater than 10-week, a clinic-based process rather than assertive follow-up, offer plausible explanations. Literature reporting adverse effects for both Varenicline and Bupropion have consisted of uncontrolled case reports with unconfirmed causal links. Both pharmacological agents have proven safe and effective for assisting people with mental illness in achieving smoking cessation (26). In 2016 the FDA revised and removed mental health warnings for both medications (49).

Junior doctor attitudinal change toward smoking cessation was evident in this study. RMOs appeared to appreciate the detrimental health impacts of smoking and the importance of their role in facilitating smoking cessation, with all reporting a perceived duty of care to advise and aid patients in cutting down and quitting smoking. This is consistent with literature demonstrating increases in advice-giving by clinicians and patient quit attempts after completion of training programs (50). The value in achieving improved and sustained knowledge and clinical practice in smoking cessation was further highlighted by the reported dearth of further training opportunities in junior doctor training, outside of GP training. The increase in knowledge and improvement in confidence within RMOs was consistent with previous research showing similar results for junior medical officers within mental health, receiving training and education for the assessment and treatment of tobacco dependence (51, 52). Given the significant health burden associated with tobacco smoking worldwide, this novel clinic in a mental health setting provides a real-world and generalizable medical education and training opportunity for junior medical staff. With higher rates of smoking and lower quit rates experienced by rural and remote residents (30), this clinic offers an option to address a health discrepancy. As people with mental illness residing in rural and remote Australia are underserviced due to a “severe shortage” of consultant psychiatrists and an inclination for trainee psychiatrists to practice in urban centers (53), the Smokers’ Clinic offers an easily implementable solution to deliver smoking cessation services to this vulnerable group in remote locations. The Smokers’ Clinic initiative employs effective clinician education models (54), whilst utilizing essential strategies previously identified to overcome challenges in implementing smoking cessation programs in rural and remote settings – selection of tobacco dedicated staff; improvement in collaboration between health services; flexible access for patients; provision of subsidized pharmacotherapies; and boosting staff morale (55).

The most notable limitation to this study was the small sample size, which raises the question of generalizability – further research using a larger sample size would be beneficial. The small sample size may have resulted in the study being underpowered. Selection bias was thought to be less relevant given the favorable response rate (70%) but was considered as those RMOs who received a beneficial experience operating the Smokers’ Clinic may have been more inclined to respond. This novel approach to assessing and managing smoking cessation requires further evaluation in other mental health service settings, with the potential for application into other medical settings to target at-risk patients across a range of other medical disciplines. Future research assessing the client’s subjective experience with the clinic would be insightful and beneficial for the purposes of improvement of service delivery.

## Conclusion

This study demonstrates the utility of a novel approach in delivering education, training, building clinical expertise, and facilitating sustained clinical capacity amongst junior medical staff for smoking cessation in a rural and remote mental health setting. Confident knowledge, skills and positive attitudinal change can result from brief but supportive teaching and supervision of junior medical staff that may be applied to settings beyond a community mental health service. It offers an efficient and novel approach for mental health services to deliver smoking cessation services whilst enhancing and building capacity in the medical workforce for the future with the aim of reducing the burden of morbidity and mortality associated with tobacco smoking. The Smokers’ Clinic proved invaluable in a rural and remote setting. Given the disproportionate health outcomes for rural and remote residents, particularly those with mental illness, and the ongoing difficulties in medical workforce training and retention, it offers an innovative solution to address physical and mental health disparities within such a vulnerable group.

## Author disclosure

This manuscript has been submitted to The Royal Australian and New Zealand College of Psychiatrists (RANZCP) as a Scholarly Project (a summative assessment item for the RANZCP Fellowship Program) for NF.

## Data availability statement

The raw data supporting the conclusions of this article will be made available by the authors, without undue reservation.

## Ethics statement

The studies involving human participants were reviewed and approved by the WA Country Health Service Human Research Ethics Committee. The patients/participants provided their written informed consent to participate in this study.

## Author contributions

All authors listed have made a substantial, direct, and intellectual contribution to the work, and approved it for publication.
